# Salutatory

**Published:** 1855-06

**Authors:** 


*Salutatory.*—Two years ago we addressed the readers of the Buffalo Medical Journal in a few sentences of salutation on taking our place as junior editor. Since that time we have been steadily at work upon its pages. With this issue we commence anew, and assume the entire charge of its interests.

Knowing how strong a hold our former colleague had upon our readers, and accustomed to attribute the larger share of the success of the Journal to the learning, talent, and industry he expended upon it, we start off under our own name only, with some misgivings. Thus far, however, we have been more than gratified at the support which rallies around us. Good articles from old familiar pens, or from new and able ones, maintain the character of our original department, and leave us just space enough in the eclectic for the cream of the medical literature of the month; our subscription list thrives apace, old subscribers pay up, and new ones send in advance in goodly numbers, and altogether we sit down to our editorial labors with a cheerful spirit, and pleasant auspices for the future.

We have few promises to make. We edit this Journal because it pays. We believe that with more labor, more pains, more expense in illustration, it will pay still better. The strong argument of money, if not the only, is one of the best incentives to labor; and in the hope—nay the certainty—that the laborer shall not be without his reward, we shall strive to make this a model Journal.

We have no claims for especial support. We have little doubt that the good cause of legitimate medicine will live long after we have ceased from our labors, and while our pen shall never be wanting in its defence, we prefer to find our mission in maintaining a good journal, readable, sprightly, and intrinsically valuable, for which no man subscribes as an act of charity, or of personal favor to ourselves. When we fail to make the Journal worth its subscription price, we have no expectation of maintaining it on any other claim to support.

